# Examining the current standards for genetic discovery and replication in the era of mega-biobanks

**DOI:** 10.1038/s41467-018-07348-x

**Published:** 2018-11-29

**Authors:** J. E. Huffman

**Affiliations:** Center for Population Genomics, MAVERIC, VA Boston Healthcare System, Boston, MA 02130 USA

## Abstract

With the recent deluge of mega-biobank data, it is time to revisit what constitutes “replication” for genome-wide association studies. Many replication samples are unavailable or underpowered, therefore alternatives beyond strict statistical replication are needed until the required resources become available.

## Abstract (web summary)

Genome-wide association studies (GWAS) have become a mainstay in genetics research to understand genotype-phenotype relationships. Following the second release of UK Biobank data and the flood of publications using these data, here the author revisits the standards for discovery, replication and follow-up in GWAS today.

## Introduction

Since the first published genome-wide association study (GWAS) in 2005^1^, a guiding principle in research conduct and interpretation has been that the strength and generalizability of GWAS findings relies upon reproducibility, grounded in strong independent statistical replication. This principle was highlighted in a seminal 2007 paper by Chanock et al.^2^ (NCI-NHGRI Working Group on Replication in Association Studies) regarding reproducibility for genotype–phenotype associations. The first GWAS in 2005 contained 96 cases and 50 controls^1^, and the Chanock et al. article was published in the same Nature issue as the Wellcome Trust Case Control Consortium’s landmark study containing 14,000 cases with 3,000 common controls^3^, the largest GWAS at the time. By contrast, this year we have seen the first published GWAS of >1 million participants^4^ as data from several large mega-biobanks become available. While several recommendations from Chanock et al. continue to hold true, four specific points merit further consideration in the current era. These points focus on (1) replication sample size, (2) access to independent datasets for replication, (3) use of similar populations for replication, and (4) the rationale for selecting replication SNPs. (see Box 1) It is timely to revisit this subject in the context of the vast advances in the last 11 years, focusing on the unique challenges for replication that large mega-biobanks present due to their size, phenotype-specificity, and population diversity. In this context, we define a mega-biobank as a study with phenotype and genotype data on >100,000 individuals and the term will refer to the study, rather than to the physical sample repository. As researchers strive to achieve the largest sample sizes possible and investigate new unique phenotypes, this Comment aims to revisit the basis for strict statistical replication as a mandatory requirement for publications with discovery sample sizes in the hundreds of thousands.

**Fig. 1:**
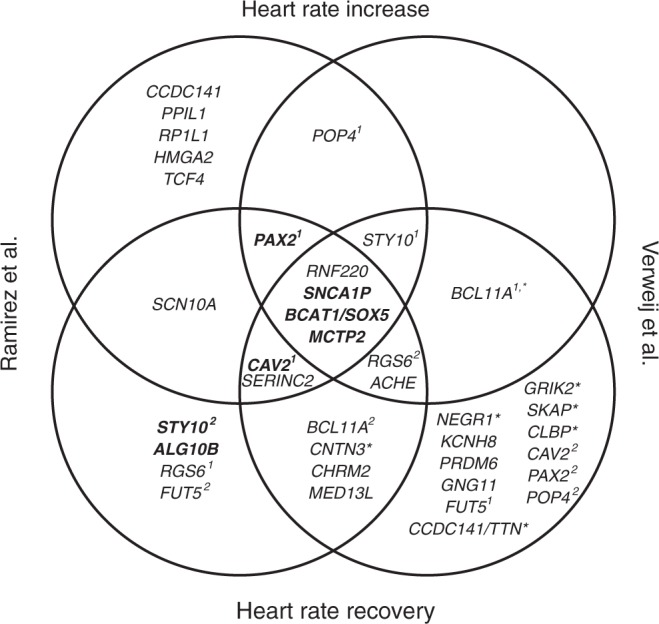
Comparison of loci discovered by each manuscript for heart rate increase or heart rate recovery in response to exercise. Heart rate increase in both manuscripts was defined in the same way (peak heart rate − resting heart rate). Heart rate recovery in Ramirez et al. was defined as peak heart rate—minimum heart rate 1 min post-exercise. Heart rate recovery in Verweij et al. was defined in the same manner at 10, 20, 30, 40, and 50 s post-exercise using mean ± 3 s. Heart rate recovery at 50 s was used for comparison as it was closest to the method used by Ramirez et al. Due to current data availability, only genes that reached genomewide significance were able to be compared. Genomewide significance in Ramirez et al. was defined as *p* < 5 × 10^–08^ and in Verweij et al. as *p* < 8.3 × 10^–09^ (corrected for the number of traits analyzed). Gene names in bold indicates locus reached genome-wide significance in the discovery data set for Ramirez et al. All others only reached significance in the full data. A superscript number after the gene name indicates independent signals based on LD (*r*^2^) calculated using 1000G phase 3 version 5 European data. *Indicates that this gene was significantly associated with a heart rate recovery measure in Verweij et al. but not at 50 s

Two recent publications in Nature Communications provide insights into a few of these issues. Verweij et al. and Ramirez et al. both report genetic variants associated with measures of heart rate response and recovery after exercise^5,6^ based on GWAS using UK Biobank data. Verweij et al. used the full dataset for discovery and did not provide replication. Ramirez et al. divided the sample into a discovery and replication set, but additionally analyzed all individuals together. A comparison of methodologies is reported in Table 1 and a comparison of locus discovery in Fig. 1. A direct comparison of results is difficult due to differing sample sizes resulting from differences in data cleaning techniques, regression models, and methodology but overall the findings presented in the two manuscripts overlapped substantially. I here consider these publications in the context of the four points mentioned above:

1. *Replication sample size*: This is the largest exercise ECG dataset including genetic data in the world and as such there is no reasonably sized external replication cohort available. This will continue to be a problem with specialized or difficult-to-measure traits, which may be available in very few individual studies. In addition, any attempts at replication would necessarily involve meta-analysis of numerous much smaller studies and therefore have decreased power.
2. *Independent datasets for replication*: While Ramirez et al. split the data into discovery and replication sets, only half of the loci which achieved genome-wide significance in the full combined dataset (discovery + replication) reached genome-wide significance in the discovery, and many of these did not surpass the modest cut-off of *p* < 1 × 10^–06^ to advance to replication. These include loci (such as *ACHE* and *CHRM2*) which were also deemed significant in Verweij et al. and had previously been associated with resting heart rate in an independent dataset^7^. While other factors may have contributed to the attenuation of significance in the discovery set, such as the use of a model adjusting for resting heart rate, these signals were present in the full dataset. Many of the loci found only in Verweij et al. were associated with heart rate recovery at earlier time points than those explored by Ramirez et al., which may explain the lack of significant association in the latter.
3. *Similar population for replication*: Despite the fact that most genome-wide studies have been conducted in populations of predominantly European ancestry (like the UK Biobank population), the unique exercise test phenotype used by these publications has not been widely conducted in other genomic studies. This further illustrates that research to study “boutique” phenotypes will continue to be problematic, although some may soon be available for extraction from electronic health record data in ongoing mega-biobank studies like the US Department of Veterans Affairs Million Veteran Program and the *All of Us Research* program. This issue is compounded in studies of non-European ancestry as there are currently few options for replication of common phenotypes, let alone rarer ones. While many new initiatives, including the *All of Us* Research Program, are underway to recruit populations that are underrepresented in biomedical research, there will be a continued GWAS publication bias due to the lack of available replication data until these new efforts are established. This bias will result from (a) lack of publication, or publication in lower tier journals since replication is often required for publication, or (b) a perceived lack of scientific rigor of these studies since replication via GWAS has become the gold standard in the field.
4. *Rationale for selecting replication SNPs*: The authors of these studies were resourceful in using available databases to further investigate regions of interest since direct GWAS replication was not available. Both studies performed conditional analyses in order to determine independent common variants to take forward for investigation and both sought evidence of association of these SNPs with correlated traits, as well as with a broad spectrum of disease outcomes. Additionally, both studies sought further supporting evidence for possible biological mechanisms by use of publically available databases to assess functional annotation, eQTL colocalization, or overlap with sites of chromatin interaction or accessibility for SNPs of interest, as well as by performance of pathway analysis. While each of these methods has its limitations, these orthogonal biological lines of evidence to explore the likelihood of association should be considered in the same vein as statistical replication.

**Table 1:** Methodology comparison between Ramirez et al. and Verweij et al. for genetic analysis of heart rate increase and recovery in response to exercise in UK Biobank.

Criteria	Ramirez et al.	Verweij et al.
Sample set	Split in to discovery (*N*~40,000) & replication (*N*~27,000) sets	Used all available data for discovery
Heart rate increase definition	Peak heart rate − resting heart rate	Peak heart rate − resting heart rate
Heart rate recovery definition	Peak heart rate—minimum heart rate 1 min post-exercise	Peak heart rate—heart rate mean at 10, 20, 30 40, or 50 s (±3 s) post-exercise
Total sample size for heart rate increase GWAS after quality control	66,800	58,818
Total sample size for heart rate recovery GWAS after quality control	66,665	58,818
GWAS software	BOLT-LMM	BOLT-LMM
Trait transformation	No transformation	Inverse-normal transformation
Covariates	Sex, age, BMI, resting heart rate, resting heart rate^2^, genotyping array	Sex, age, sex–age interaction, BMI, BMI^2^,PC1-30, genotyping array

In summary, the Ramirez et al. and Verweij et al. studies, while using the same dataset, provide different insights into the genetics governing heart rate response to, and recovery after, exercise. Due to their differing phenotype definition and modeling, different questions are answered. Ramirez et al. accounted for resting heart rate, therefore may find signals that are more specific to exercise in general, whereas the multiple time-points investigated by Verweij et al. provide insight into what genes may be important at different stages in recovery post-exercise. In addition to addressing questions about replication strategies in mega-biobanks, these studies also give insight into the opportunities for having multiple researchers tackle similar question in publically available data, since each team will have their own approach to data cleaning, analysis, and interpretation, which can be complementary.

Ultimately, GWAS findings are hypotheses generating, providing strong evidence for statistical correlation but not causation; therefore functional and interventional studies in animal models and humans will always be required to determine biological mechanisms. With the sample sizes generated by these large mega-biobanks, in combination with the rapid development of large publically available functional data, for common variants we may have moved beyond the era where strict statistical replication via GWAS is always required for publication, and additional sources of information may be taken into account when prioritizing loci for further study. This is not to say that replication should not be sought; however, while evidence is awaited from appropriately powered, diverse cohorts to become available, this may be an interim silver standard solution. Rare variants present their own challenges for replication and should be treated with greater caution so we do not revert back to the many false positive associations reported during the “candidate gene” era that sparked the Chanock et al. paper.

In addition to a call for larger study populations focused on traditionally underrepresented populations, I would also advocate for greater integration of the excellent functional databases and tools, as well as further collaboration and crosstalk between statistical/population geneticists and molecular biology scientists to dig further into underlying biological mechanisms. In the 11 years since the Chanock et al. paper, there have not only been striking advances in the population genomic data available, but also in the sensitivity and specificity of wet-lab techniques to investigate specific variants, genes, and tissues, complimented by an explosion in the catalog of available functional databases. With the integration of these amazing resources into our research pipeline, who knows what discoveries the next decade will bring.

### Box 1 Discussion of points to revisit from Chanock et al. in the context of mega-biobanks

1. *“Replication studies should be of sufficient sample size to convincingly distinguish the proposed effect from no effect”*. Determination of the proposed effect may become difficult if the discovery population consists of >500,000 individuals, particularly if the variant to be replicated is rare. In addition, achieving a sufficiently large replication sample may require a meta-analysis of many smaller studies with an accompanying decrease in power due to population heterogeneity in sample make-up and phenotyping methods. Finally, since each mega-biobank was designed independently, there are some study phenotypes that are not available in large numbers in other studies.
2. *“Replication should preferably be conducted in independent data sets to avoid the tendency to split one well-powered study into two less conclusive ones”*. While large mega-biobanks are well-powered to discover common variant associations even when split into a discovery and replication set, they offer an additional advantage in the power they afford to discover rare variant associations. Such associations may be difficult to discover and replicate using split data sets. Also, although genetic data may be split into discovery and replication sets prior to association analysis, the phenotype and genotype data will have been collected, processed, and quality controlled together, therefore it can be argued that it is not a truly independent replication set.
3. *“A similar population should be studied and notable differences between the populations studied in the initial and attempted replication studies should be described”*. Recent reports have highlighted the pressing need for genome-wide studies to focus on more diverse participants^8^. Many of the large mega-biobanks are population-specific, for example UK Biobank^9^ is largely white British (European descent), BioBank Japan^10^ contains Japanese individuals, and the Million Veteran Program^11^ is mainly male, and contains, in addition to participants of European descent, large numbers of African Americans, and Hispanic Americans. Despite the large sample sizes of mega-biobanks, this heterogeneity in itself can create issues for replication, particularly in studies seeking to replicate findings from similar non-European populations.
4. *“A strong rationale should be provided for selecting SNPs to be replicated from the initial study, including linkage-disequilibrium structure, putative functional data or published literature.”* While some recent papers have addressed significance thresholds for use in large updated imputation panels and sequencing projects, it is not immediately clear what threshold should be used for rare variants or for admixed populations, where the linkage-disequilibrium thresholds may be very different from the white, common variant data which we are used to studying. Until now, *p* < 5 × 10^–08^ has been accepted as the genome-wide threshold for significance^12,13^. Recently, papers have suggested thresholds from *p* < 1 × 10^–08^ to *p* < 1 × 10^–09^ based on method of genotype ascertainment, genetic ancestry, and variant frequency^14,15^. Neither addressed this question in the context of very large sample size, like those observed in large mega-biobanks. Additionally, the impact of each variant is not fully understood, particularly if they have a regulatory effect on the surrounding genic landscape. Even if an association can be assigned to a gene, functional information may not be readily available for all genes or may be incomplete. Therefore, lack of functional information may not be the best criteria for moving a variant forward for replication.
